# Existential concerns and fear of recurrence or progression in people with migraine

**DOI:** 10.1177/13591053261435786

**Published:** 2026-03-30

**Authors:** Vedika Yadav, Rachel E. Menzies

**Affiliations:** 1 School of Psychology, The University of Sydney, NSW, Australia

**Keywords:** migraine, fear of recurrence or progression, existential concerns, disability, death anxiety, meaninglessness

## Abstract

The current study examined the relevance and potential associations between fear of recurrence or progression (FRP) and existential concerns (i.e. death anxiety, meaninglessness, isolation, identity, and guilt) in the context of migraine. Cross-sectional survey data was obtained from 522 people with a self-reported migraine diagnosis (94% female; *M*_age_ = 42.79 years), who completed a number of online questionnaires assessing FRP, existential concerns, disability, pain, and mental health symptoms. The results revealed significant positive correlations between all five existential concern domains and FRP. Notably, the associations between each existential concern and FRP remained significant after controlling for physical symptoms (i.e. disability and pain) and psychopathology (i.e. depression, anxiety, and stress). Further, the relationship between disability and FRP was partially mediated by overall existential concerns. These findings emphasise the need to consider existential concerns in the conceptualisation and treatment of FRP in people with migraine, paving the way for future intervention research.

## Introduction

Migraine is a disabling neurological condition that affects approximately 12% of the global population (Fan et al., 2023; Woldeamanuel and Cowan, 2016), characterised by recurrent headaches of moderate to severe intensity, sufficient to limit daily activities (Headache Classification Committee of the International Headache Society (IHS), 2018). Migraine disorders are often accompanied by symptoms such as nausea, fatigue, vomiting and aura (i.e. sensory disturbances; Bigal and Lipton, 2008). The GBD 2016 Headache Collaborators (2018) ranked migraine as the third highest cause of disability worldwide, with the condition frequently associated with several life-limiting medical comorbidities (e.g. epilepsy, sleep and inflammatory disorders, cardiovascular diseases; Burch et al., 2019). In addition to the physical impact of the condition, individuals with migraine report greater levels of depression, anxiety, and stress than those without (Jette et al., 2008; Lantéri-Minet et al., 2005; Wacogne et al., 2003). Despite these facts, migraine remains one of the most understudied and underdiagnosed medical conditions globally (Wijeratne et al., 2019).

Considering the high burden of disease experienced by people with migraine, it is perhaps unsurprising that these individuals may report fears related to their disorder. Existing research has revealed that those with migraine commonly report experiencing anticipatory anxiety about future migraine attacks (Estave et al., 2021; Martelletti et al., 2018). Recently, such illness-specific concerns have been investigated under the construct of fear of progression (FoP), defined as “the fear that the illness will progress with all its biopsychosocial consequences, or that it will recur” (Herschbach and Dinkel, 2014: 13) Prior to the inception of FoP, fears in the context of ill health were typically studied as disease-specific manifestations of anxiety, such as the fear of cancer recurrence (FCR; Herschbach et al., 2005). As research into FCR has grown, it has become apparent that such concerns involving illness recurrence and progression are not exclusive to the experience of cancer, with FoP being observed across various disorders, including rheumatoid arthritis, diabetes mellitus, multiple sclerosis (Herschbach and Dinkel, 2014), endometriosis (Pickup et al., 2024), and even COVID-19 (Ding et al., 2022).

In noting the parallels between FCR and FoP, emerging research has questioned whether they are the same construct. Coutts-Bain et al. (2022) compared the two constructs in a sample of women with cancer, finding that they were distinct yet strongly related, and shared overlapping predictors. More recently, Sharpe et al. (2024) developed a transdiagnostic and unidimensional measure of fear of recurrence or progression (FRP): the Worries about Recurrence or Progression Scale (WARPS). By comparing results from a range of different illness groups (e.g. respiratory, rhematic, diabetic, and cardiovascular diseases), the findings suggested that FRP may be a single, overarching construct (Sharpe et al., 2024). Given that migraine is characterised by recurrent attacks and has the potential to progress in both frequency and intensity (Bigal and Lipton, 2008; Buse et al., 2009), one might expect that FRP would be especially pertinent to the migraine experience. However, this relationship has not yet been investigated.

Additionally, it is essential to examine what variables might play a key role in FRP. In this vein, five existential concerns have been previously linked to FRP in the context of other physical conditions (Curran et al., 2020; Sharpe et al., 2023a, 2023b, 2024). Yalom (1980) and Koole et al. (2006) argue that individuals may grapple with five “givens” unique to human existence. These concerns include: (1) the desire to live, whilst cognisant of the inevitability of death (*death anxiety*), (2) a need to believe life is meaningful, whilst existing in a world that appears to have no inherent order or purpose (*meaninglessness*), (3) the struggle to find a clear and cohesive sense of self which conflicts with the uncertainties of one’s innermost character (*identity*), (4) a desire to feel connected to others, whilst knowing that one’s subjective experience can never by fully shared or understood (*isolation*), and (5) a need to make choices in life amidst the various options available to us, and the resulting guilt which can emerge from choosing poorly (*guilt*). These existential concerns are not assumed to be static or uniformly experienced. Instead, their presence or salience may differ markedly across individuals, contexts, and time (Yalom, 1980).

Why might the five existential concerns be particularly relevant to FRP in migraine? First, migraine can significantly limit a person’s ability to socialise, work or pursue meaningful goals (Atari, 2019; Lipton et al., 2003). As a result, an individual’s concerns about their symptoms recurring or worsening may, in part, stem from reduced opportunities to find purpose in life, and difficulty continuing to engage in activities which shaped one’s pre-illness identity. Similarly, those who experience guilt over past life decisions and former inaction, or feel dread about their potentially limited future, may be more fearful about the prospective course of their condition (Arian et al., 2023). In addition, people with migraine often report feeling misunderstood by others due to the invisible nature of the illness (Rutberg and Öhrling, 2012). This experience of feeling a “gap” between oneself and others underlies existential isolation (Yalom, 1980), which may thereby contribute to greater FRP. Lastly, it is possible that for some individuals, FRP may arise from catastrophic interpretations of migraine symptoms (e.g. interpreting severe head pain or aura as signs of a brain tumour or stroke). Thus, while migraine is not a fatal condition, death anxiety may be a contributing factor to the fear of migraine symptoms returning or worsening.

Whilst the five existential concerns have not previously been explored in the context of migraine, they have been argued to play a role in FRP in other chronic illnesses. Simonelli et al. (2017) assert that receiving a cancer diagnosis can result in an existential crisis, giving rise to death anxiety and challenging the individual’s ability to make meaning. Recent research has revealed that death anxiety is an important predictor of FCR amongst cancer patients (Curran et al., 2020) and is strongly associated with FoP, even in diseases which were not life-threatening (Sharpe et al., 2024). However, at present, only one study has explicitly measured the relationships between all five of the aforementioned existential concerns and FoP in people with chronic illness. Here, Sharpe et al. (2023b) found that FoP was significantly correlated with existential concerns in individuals with rheumatoid arthritis. Importantly, results indicated that the concerns of death anxiety, guilt, and meaninglessness contributed unique variance to FoP even after controlling for all other variables. Such findings highlight the importance of existential issues, not only in regards to conceptualising fears of illness recurrence or progression, but also in revealing target areas for intervention.

### The Current Study

The current study aimed to address several limitations in the existing literature. Despite their theoretical relevance, no studies have explicitly measured FRP or the five existential concerns (i.e. death anxiety, meaninglessness, identity, isolation, and guilt) amongst individuals with migraine. As such, this study sought to evaluate the levels and relevance of these constructs, as well as their relationship in people with migraine. We hypothesised that higher levels of existential concerns amongst individuals with migraine would be associated with greater FRP. Moreover, the present research aimed to establish whether each existential concern explained unique variance in FRP, whilst controlling for physical symptoms (i.e. disability and pain) and psychopathology (i.e. depression, anxiety, and stress). Additionally, considering the high levels of functional impairment reported by individuals with migraine, we hypothesised that existential concerns would partially mediate the effect of disability on FRP.

## Method

### Participants

An a priori power analysis was conducted using G*Power for multiple linear regression (Faul et al., 2007). This indicated that 143 participants would be needed to provide 80% power to detect a small-to-medium effect size (Cohen’s *f*^2^ = 0.1) at α = 0.05, with six tested predictors. This calculation was based on those found in previous studies with similar methodologies and hypotheses (Pickup et al., 2024; Wang et al., 2022).

We recruited participants through advertisement in social media groups (e.g. Facebook and Instagram), websites of relevant organisations (e.g. Migraine and Headache Australia), and relevant local clinics (e.g. Sydney Headache and Migraine Clinic). To be eligible for the study, participants had to: (1) be 18 years or older; (2) be proficient in English; and (3) report having experienced at least one migraine in the last 6 months that was not associated with another illness, head injury, or hangover. Ethics approval was obtained from the Human Research Ethics Committee of the University of Sydney, Australia (#2024/HE000596), and all procedures were conducted in accordance with the national ethical guidelines. The study was also pre-registered on Open Science Framework (https://osf.io/2br56).

### Procedure

Upon expressing interest in the study, respondents were directed to an online questionnaire. Participants who provided written informed consent and met the eligibility criteria were granted access to the survey which comprised the measures outlined below.

### Measures

#### Demographic questionnaires

Demographic questionnaires assessed participant age, gender, highest level of education, relationship status, ethnicity, and country of residence. In addition, participants reported their age at first migraine, for how many years they experienced migraine, and method of diagnosis.

#### Worries About Recurrence or Progression Scale (WARPS)

The WARPS (Sharpe et al., 2024) is an 18-item questionnaire that was developed as a transdiagnostic measure assessing fears about recurrence or progression across various physical illnesses (e.g. “It worries me that my illness could suddenly return or progress”). In the current study, participants were instructed to respond with reference to their migraine. Items are rated on a 5-point Likert scale ranging from 1 (“Strongly disagree”) to 5 (“Strongly agree”), with a higher score indicating greater FRP. The WARPS has been validated in patients from multiple illness groups, as well as in a large cancer sample, demonstrating excellent validity and reliability (Sharpe et al., 2024; Smith et al., 2024). In the current sample, the internal consistency was excellent (α = 0.97).

#### Existential Concerns Questionnaire (ECQ)

The ECQ (van Bruggen et al., 2017) is a 22-item scale measuring five existential concern domains: Death anxiety (seven items; e.g. “It frightens me at some point in time I will be dead”; α = 0.89), Meaninglessness (four items; e.g. “I worry about the meaning of life”; α = 0.82), Isolation (three items; e.g. “I have the anxious feeling that there is a gap between me and other people”; α = 0.79), Identity (four items; e.g. “I try to avoid the question of who I really am”; α = 0.84), and Guilt (four items; e.g. “It frightens me when I realise how many choices life offers,” α = 0.77). Response options range from 1 (“Never”) to 5 (“Always”), where higher scores indicate greater existential concerns. The use of separate scores representing each existential concern has been supported by previous research demonstrating their adequate psychometric properties (Chawla et al., 2022; Sharpe et al., 2023b). The ECQ has demonstrated good construct and content validity, as well as test-retest reliability across diverse clinical and non-clinical samples (García-Alandete, 2025; van Bruggen et al., 2017). In the current study, the internal consistency for the overall scale was excellent (α = 0.95).

#### Migraine Disability Assessment Questionnaire (MIDAS)

The MIDAS (Stewart et al., 2001) is a five-item, self-report questionnaire which records the number of days in the past 3 months in which headache symptoms caused missed or reduced activity across three domains: employment or school-work; household work and family; and social or leisure activities. Summed MIDAS scores are graded on severity from none or little (0–5), mild (6–10), moderate (11–20) to severe disability (≥21). The reliability, validity, and clinical utility of the MIDAS have been widely documented in migraine patient samples across cultures, demonstrating good psychometric properties (Stewart et al., 2000, 2001; Zandifar et al., 2014). In the present study, the internal consistency for the five-item MIDAS was good (α = 0.86).

##### Migraine frequency and pain

Two additional items accompany the MIDAS to collect information on migraine frequency (“On how many days in the last three months did you have a migraine?”) and pain intensity (“On a scale of 1-10, on average how painful were these migraines?”). These additional MIDAS items have exhibited good psychometric properties (Kwong and Pathak, 2007).

#### Depression, Anxiety, and Stress Scale-21 (DASS-21)

The DASS-21 (Lovibond and Lovibond, 1995) assesses: Depression (seven items; e.g. “I felt down-hearted and blue”), Anxiety (seven items; e.g. “I felt scared without any good reason”), and Stress (seven items; e.g. “I found it difficult to relax”). Participants rate the extent to which each item applied to them over the last week on a 4-point rating scale ranging from 0 (“Never”) to 3 (“Almost always”). Higher scores signify greater severity of psychological distress, with symptom severity for each subscale interpreted as normal, mild, moderate, severe, or extremely severe. Each subscale yields a score ranging from 0 to 21, and the reliability and validity of the DASS-21 are well established (Henry and Crawford, 2005). In the current sample, the internal consistency for the three subscales ranged from good (α = 0.83) to excellent (α = 0.91).

### Planned Statistical Analysis

Prior to conducting the main analyses, the data was screened for failures on two instruction-based attention checks, and participants who failed both checks were excluded. To test the first hypothesis, we calculated Pearson correlations between WARPS scores and each of the five subscales of the ECQ, as well as disability, pain, depression, anxiety, and stress. Additionally, a series of hierarchical regression analyses were conducted to determine which, if any, of the five ECQ domains accounted for unique variance in FRP. The variables of disability, pain, depression, anxiety, and stress were entered into the first step. To assess whether existential concerns were associated with FRP over and above these psychosocial variables, the total and subscale ECQ scores were entered in the second step. Finally, to examine the second hypothesis, we used the Hayes (2014) PROCESS macro (Model 4) in SPSS to investigate the potential mediation effects of existential concerns on the relationship between disability and FRP. A *p*-value of <0.05 was considered statistically significant.

## Results

### Participant and Sample Characteristics

A total of 565 people responded to the study advertisement, with 527 (93.3%) of these providing informed consent. Of those responses, five participants failed both attention checks and were excluded from the analysis. Of the remaining 522 participants, 418 (80.1%) reached the end of the survey. For participants who discontinued the survey prior to completion, their data was included up to the final measure they responded to.

On average, participants were 42.79 years old (SD = 11.67), reported experiencing their first migraine at 18.99 years (SD = 10.70), and had lived with migraine for approximately 22.22 years (SD = 13.74). Further demographic and migraine diagnostic information are shown in Table 1. Descriptive statistics for the study measures are shown in Table 2.

**Table 1.** Frequencies of demographic and migraine diagnostic information.

	*n*	%
Gender		
Female	472	94.0
Male	26	5.2
Non-binary/Gender non-conforming	3	0.6
Other	1	0.2
Education		
Did not complete high school	16	3.2
Completed high school	55	11.0
Completed diploma or equivalent certificate	137	27.3
Completed undergraduate degree	170	33.9
Completed postgraduate degree	124	24.7
Relationship status		
Single	105	20.7
In a relationship	76	14.9
De facto	45	8.9
Married	258	50.9
Divorced	19	3.7
Widowed	4	0.8
Ethnicity		
White	424	84.5
Asian	40	8.0
African	2	0.4
Aboriginal or Torres Strait Islander	5	1.0
Middle Eastern	8	1.6
Other	23	4.6
Country of residence		
Australia	432	86.1
USA	28	5.6
United Kingdom	8	1.6
Other	34	6.8
Method of diagnosis		
General practitioner	229	46.6
Neurologist	192	39.1
Self-diagnosed	45	9.2
Other	25	5.1

**Table 2.** Descriptive statistics of study measures.

Measure	*N*	Min.	Max.	*M*	SD
WARPS	472	18	90	64.46	17.90
ECQ-Total	449	22	102	50.12	18.94
ECQ-Death Anxiety	449	7	35	16.62	6.83
ECQ-Guilt	449	4	20	10.09	3.69
ECQ-Meaninglessness	449	4	20	8.44	3.74
ECQ-Isolation	449	3	15	6.94	3.27
ECQ-Identity	449	4	20	8.03	3.89
MIDAS	427	0	465	69.70	79.39
Pain	413	1	10	6.85	1.68
DASS-21-Depression	418	0	21	6.66	4.97
DASS-21-Anxiety	418	0	20	5.25	4.14
DASS-21-Stress	418	0	21	8.86	4.37

*Note*. WARPS: Worries About Recurrence or Progression Scale; ECQ: Existential Concerns Questionnaire; MIDAS: Migraine Disability Assessment Questionnaire; DASS-21: Depression, Anxiety, Stress Scale-21.

### Primary Analyses

#### Correlations between FRP, existential concerns and psychosocial variables

As shown in Table 3, FRP (i.e. total WARPS score) was positively and significantly correlated with disability, pain, depression, anxiety, and stress. There was also a significant, moderate correlation found between FRP and total ECQ scores, as well as with each of the five ECQ subscales. Additionally, Fisher *r*-to-*z* transformations were conducted to determine whether there were any significant differences in the size of the correlations between each existential concern and WARPS scores. This revealed that none of these correlations were significantly different to one another (all *p*s > 0.201), suggesting that the size of the relationships with FRP were largely equivalent across each of the five existential concerns.

**Table 3.** Pearson correlations between fear of recurrence or progression (WARPS) and other variables.

Measure	1	2	3	4	5	6	7	8	9	10	11	12	13
1. WARPS	-												
2. ECQ-Total	0.49**	-											
3. ECQ-Death Anxiety	0.45**	0.89**	-										
4. ECQ-Guilt	0.46**	0.88**	0.70**	-									
5. ECQ-Meaninglessness	0.43**	0.89**	0.72**	0.77**	-								
6. ECQ-Isolation	0.42**	0.87**	0.68**	0.75**	0.73**	-							
7. ECQ-Identity	0.39**	0.88**	0.66**	0.76**	0.77**	0.81**	-						
8. MIDAS	0.38**	0.22**	0.17**	0.22**	0.21**	0.27**	0.16**	-					
9. Pain	0.24**	0.14**	0.09	0.15**	0.15**	0.11*	0.14**	0.13*	-				
10. DASS-21-Depression	0.47**	0.69**	0.53**	0.65**	0.63**	0.66**	0.67**	0.33**	0.20**	-			
11. DASS-21-Anxiety	0.43**	0.58**	0.52**	0.51**	0.44**	0.53**	0.59**	0.21**	0.16**	0.56**	-		
12. DASS-21-Stress	0.41**	0.56**	0.52**	0.49**	0.42**	0.49**	0.54**	0.09	0.16**	0.59**	0.67**	-	
13. Migraine Frequency	0.31**	0.15**	0.09	0.16**	0.15**	0.19**	0.13*	0.67**	−0.04	0.25**	0.12*	0.06	-

*Note*. WARPS: Worries About Recurrence or Progression Scale; ECQ: Existential Concerns Questionnaire; MIDAS: Migraine Disability Assessment Questionnaire; DASS-21: Depression, Anxiety, Stress Scale-21.

* *p* < 0.05 (two-tailed). **p < 0.01 (two-tailed).

#### Predictors of FRP

Next, six hierarchical regression analyses were conducted to assess whether the association between existential concerns and FRP in people with migraine remained significant whilst controlling for physical symptoms (i.e. disability and pain) and psychopathology (i.e. depression, anxiety, and stress). These latter variables were entered in the first step, with the total WARPS score as the dependent variable. Together, disability, pain, depression, anxiety, and stress accounted for 33.0% of the variance in FRP.

Following this, the ECQ total score and each of the ECQ subscales were entered into the second step. The results of these six hierarchical regressions indicated that total ECQ scores, and each of the five subscales were significantly associated with FRP over and above physical symptoms and psychopathology (see Table 4).

**Table 4.** Results of six hierarchical regressions with fear of recurrence or progression (WARPS) as the dependent variable (*n* = 404).

		Model 1					Model 2				
		*B*	SE *B*	*β*	*p*	*R* ^2^	*B*	SE *B*	*β*	*p*	*R* ^2^
(1)	MIDAS	0.05	0.01	0.25	<0.001	0.33	0.05	0.01	0.24	<0.001	0.37
	Pain	1.18	0.43	0.11	0.007		1.22	0.42	0.12	0.004	
	Depression	0.74	0.20	0.21	<0.001		0.25	0.22	0.07	0.240	
	Anxiety	0.57	0.24	0.13	0.019		0.29	0.24	0.07	0.230	
	Stress	0.62	0.24	0.15	0.010		0.48	0.24	0.12	0.041	
	ECQ-Total						0.26	0.05	0.28	<0.001	
(2)	MIDAS						0.05	0.01	0.24	<0.001	0.36
	Pain						1.26	0.42	0.12	0.003	
	Depression						0.50	0.20	0.14	0.012	
	Anxiety						0.35	0.24	0.08	0.143	
	Stress						0.43	0.24	0.11	0.074	
	Death Anxiety						0.59	0.13	0.23	<0.001	
(3)	MIDAS						0.05	0.01	0.24	<0.001	0.37
	Pain						1.14	0.42	0.11	0.007	
	Depression						0.28	0.21	0.08	0.191	
	Anxiety						0.35	0.24	0.08	0.139	
	Stress						0.54	0.23	0.13	0.022	
	Guilt						1.27	0.25	0.27	<0.001	
(4)	MIDAS						0.05	0.01	0.24	<0.001	0.36
	Pain						1.14	0.43	0.11	0.008	
	Depression						0.35	0.22	0.10	0.107	
	Anxiety						0.45	0.24	0.11	0.056	
	Stress						0.60	0.24	0.15	0.010	
	Meaninglessness						0.96	0.24	0.21	<0.001	
(5)	MIDAS						0.05	0.01	0.24	<0.001	0.34
	Pain						1.22	0.43	0.12	0.005	
	Depression						0.50	0.22	0.14	0.021	
	Anxiety						0.45	0.24	0.11	0.068	
	Stress						0.58	0.24	0.14	0.016	
	Isolation						0.74	0.30	0.14	0.014	
(6)	MIDAS						0.06	0.01	0.25	<0.001	0.34
	Pain						1.19	0.43	0.12	0.006	
	Depression						0.51	0.22	0.15	0.020	
	Anxiety						0.42	0.25	0.10	0.094	
	Stress						0.58	0.24	0.14	0.016	
	Identity						0.57	0.26	0.13	0.028	

*Note*. *B*: unstandardised beta; SE B: standard error of *B*; *β*: standardised beta; ECQ: Existential Concerns Questionnaire; MIDAS: Migraine Disability Assessment Questionnaire; WARPS: Worries About Recurrence or Progression Scale.

#### Mediating role of existential concerns in the relationship between FRP and disability

A mediation analysis tested whether the significant relationship observed between disability and FRP was in fact mediated by overall existential concerns. The MIDAS score was entered as the independent variable, ECQ Total score was entered as the mediator, and WARPS score was entered as the dependent variable.

In Step 1 of the mediation model, the regression of disability on FRP, without including existential concerns (i.e. the mediator), was significant, *b* = 0.084, *p* < 0.001. Step 2 showed that the regression of disability on existential concerns was also significant, *b* = 0.053, *p* < 0.001. Next, Step 3 of the mediation process indicated that existential concerns, controlling for disability, significantly predicted FRP, *b* = 0.403, *p* < 0.001. Step 4 of the analyses revealed that, controlling for existential concerns, disability was a significant predictor of FRP, *b* = 0.063, *p* < 0.001 (see Figure 1).

**Figure 1.**
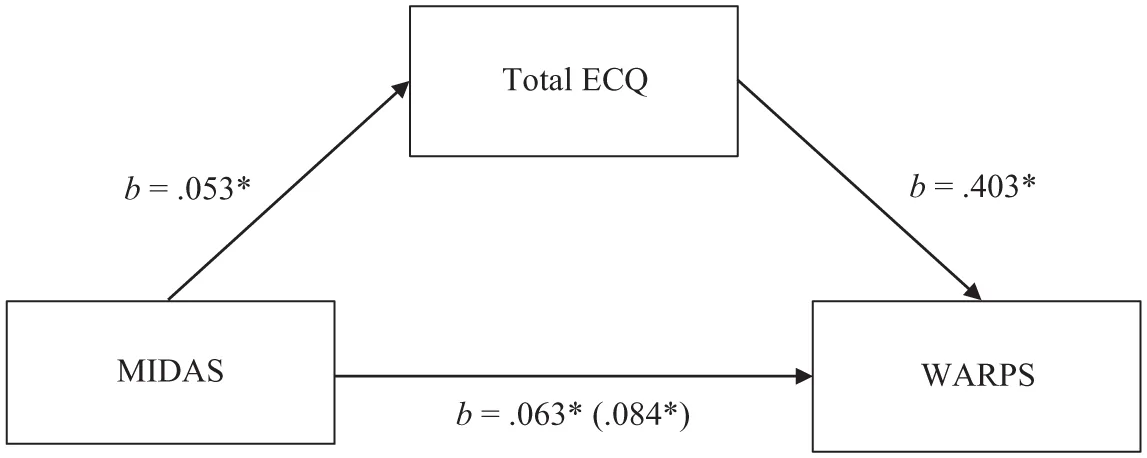
Mediation Model between Disability (MIDAS) and Fear of Recurrence or Progression (WARPS), with Existential Concerns (ECQ) as the Mediator (*n* = 428). *Note*. Model is testing the mediation of existential concerns on the relationship between disability and fear of recurrence or progression. *b*: unstandardised beta coefficient; ECQ: Existential Concerns Questionnaire; MIDAS: Migraine Disability Assessment Questionnaire; WARPS: Worries About Recurrence or Progression Scale. *p < 0.001.

These results indicate that greater disability was associated with greater existential concerns, which in turn resulted in higher FRP. Despite this support for the mediation, the direct effect of disability on FRP remained significant. Therefore, the model suggested a partial mediation, with a significant indirect effect of disability on FRP through overall existential concerns (*b* = 0.021, SE = 0.005, 95% CI [0.012, 0.032]).

## Discussion

The present study aimed to investigate whether existential concerns were associated with FRP in people living with migraine. All five existential concerns, as well as disability, pain, depression, anxiety, and stress were moderately correlated with FRP. Notably, the relationships between FRP and each individual existential issue remained significant after controlling for physical symptoms (i.e. disability and pain) and psychopathology (i.e. depression, anxiety, and stress). Further, consistent with predictions, overall existential concerns were found to partially mediate the relationship between disability and FRP in people with migraine. In other words, individuals who are more disabled by their migraine may, in turn, be more susceptible to issues of meaninglessness, isolation, identity, guilt, and death anxiety, which may consequently amplify their FRP.

The current findings underscore the relevance of FRP amongst those experiencing migraine. Interestingly, the average FRP scores found in the current investigation closely mirror those reported by individuals experiencing more overtly life-threatening illnesses such as cardiovascular diseases (Sharpe et al., 2024). One explanation for this similarity could be that disability, rather than a direct threat to life, contributes to such illness-related worries in people with migraine. Supporting this, the Global Burden of Diseases study (Abate et al., 2017) found that patients with migraine experienced a greater number of disability-adjusted life years than those with hypertensive heart disease, cardiomyopathy, and atrial fibrillation. The current finding that FRP is relevant to migraine extends previous research linking illness-related fears with other physical conditions such as cancer, rheumatoid arthritis, and diabetes (Herschbach and Dinkel, 2014; Michalski et al., 2024; Sharpe et al., 2023b).

The current study is the first to demonstrate that each of the five existential concerns are associated with FRP in migraine. First, the existential concern of meaninglessness was found to contribute independent variance to FRP in people with migraine, such that a sense that one’s life lacked purpose was associated with greater worries about the future course of one’s migraine. This result is understandable given that this population often report diminished engagement in social, occupational, and other valued pursuits (Leonardi and Raggi, 2019) which may affect their ability to search for and construct meaning in life. Whilst Curran et al. (2020) found that meaning in life did not predict FoP in cancer patients, Sharpe et al. (2023b) reported that meaninglessness uniquely contributed to FoP in individuals with rheumatoid arthritis.

Existential isolation also emerged as a significant predictor of FRP. From this finding, it appears that those who have heightened feelings of a “gap” between the self and others, or feel that other people may never completely understand their subjective experience, may be more anxious about the potential recurrence or progression of their illness over time. This could be due to people with migraine having a decreased capacity to partake in socio-familial interactions (Bigal et al., 2006), feelings of not being believed due to the condition’s invisible character (Rutberg and Öhrling, 2012), and misconceptions that their condition is “just a headache” (Parikh et al., 2021; Shapiro et al., 2025). Interestingly, although existential isolation appears theoretically relevant to experiences of chronic illness, research in other diseases (e.g. cancer, rheumatoid arthritis) has not demonstrated a strong association with FoP (Li et al., 2023; Sharpe et al., 2023b). The current findings therefore highlight the potentially unique role of existential isolation in migraine, arguably stemming from the stigma and limited public awareness of the condition, given its non-fatal, invisible, and episodic nature (Parikh et al., 2021; Shapiro et al., 2025).

Furthermore, our findings emphasise the role of existential guilt in illness-related fears, with this domain found to be significantly associated with FRP over and above the psychosocial variables. In other words, consistent with prior research in rheumatoid arthritis (Sharpe et al., 2023b), those who feel guilt over their former choices (e.g. not having used one’s time wisely), or feel worried that they may no longer be able to live life to its fullest, may be more fearful about the future trajectory of their illness. This result is understandable as individuals with migraine often experience highly disabling symptoms that can adversely impact their ability to participate in day-to-day affairs and plan for the future (Burch et al., 2019; Leonardi and Raggi, 2019). Consequently, these impediments could contribute to negative interpretations about one’s past and future life choices (i.e. existential guilt).

Identity also contributed independently to FRP, such that a greater degree of uncertainty about one’s identity was associated with more anxiety about the course and consequences of one’s migraine. This may be due to reduced functioning in social, professional, and familial life domains amongst people with migraine (Buse et al., 2009; Leonardi and Raggi, 2019) which could disrupt or threaten an individual’s self-concept and pre-illness identity. To date, identity has received little attention with regard to illness-related fears, and, interestingly, was not found to be significantly associated with FRP in the context of rheumatoid arthritis (Sharpe et al., 2023b). It is possible that identity may be particularly relevant in migraine due to the unique stigmatising stereotypes associated with this disease, including cultural perceptions of those with migraine as weak, lazy, and highly-strung (Parikh et al., 2021). Such stereotypes may negatively impact one’s self-perception and heighten concerns about the future course of one’s illness.

Lastly, the existential domain of death anxiety was observed to be a significant predictor of FRP in the present sample. That is, worries about death appear relevant to concerns about the recurrence or progression of one’s illness, aligning with previous findings in other chronic conditions (Berlin and von Blanckenburg, 2022; Curran et al., 2020; Sharpe et al., 2023a, 2024). One potential explanation for this positive association is that individuals with migraine may interpret their physiological symptoms such as severe head pain, nausea, muscle weakness, and aura as signs of illness deterioration, or even fatal disease (e.g. stroke, brain tumour). In fact, one prior study indicated that a brain tumour was the most common fear reported by people experiencing migraine in the context of their illness, outnumbering fears of work loss, pain, and interference with social activities (Blau, 1984). This finding suggest that the migraine symptoms may trigger mortality concerns, in turn heightening FRP. Similarly, experimental evidence has demonstrated the causal role of death anxiety in driving maladaptive behaviours relevant to FRP (e.g. increased time spent monitoring one’s body for symptoms; Menzies et al., 2021). Importantly, the items assessing death anxiety used in this study contained no references to illness or disease, yet still predicted FRP. This suggests that broader existential concerns related to mortality and non-existence, rather than illness-specific anxiety alone, may play a role in shaping these fears. However, future research is needed to further elucidate the association between death anxiety and FRP in migraine, particularly given the non-fatal nature of the disorder.

### Strengths and Limitations

This study has several noteworthy strengths. First, potential confounds (e.g. disability, pain, general anxiety) were controlled for in the current analyses, which may raise confidence in the robustness of these findings. Additional strengths include study pre-registration and the use of a large, sufficiently powered sample. Importantly, the selection of a transdiagnostic measure to assess FRP allowed for direct comparisons with other chronic illnesses such as rheumatic, respiratory, and cardiovascular diseases.

Despite the useful contributions of the current research, it is not without limitations. Most notably, the cross-sectional design of the study precludes conclusions regarding causality. Although it is possible that existential concerns heighten a person’s fears about their illness recurring or progressing, it is equally possible that experiencing FRP leads to greater levels of existential concerns. Whilst Simonelli et al. (2017) speculate that existential concerns (i.e. death anxiety) are causally important to FCR, no empirical studies have demonstrated the direction of this relationship. Experimental and longitudinal research is required to further examine the direction of causality in the associations between existential concerns and FRP.

Another limitation of this study is that the current sample comprised of participants who self-reported their migraine diagnosis. As such, it is unclear whether the current findings would replicate in people who were screened for migraine by a trained clinician. However, the reported levels of disability (i.e. MIDAS scores) and pain in this study were largely equivalent to those found in previous studies using participants whose migraine diagnosis was confirmed by a neurologist (Renjith et al., 2016; Stewart et al., 2000). Nevertheless, future studies should aim to replicate these findings using participants with medically confirmed migraine diagnoses.

Finally, the sample did not accurately reflect the gendered prevalence of migraine, which epidemiological data indicate is approximately three times higher in females than in males (Delaruelle et al., 2018; Fan et al., 2023). Instead, the present sample had a substantially higher rate of women (94.0%) relative to the epidemiological prevalence of migraine, with only 5.2% of participants identifying as male. As a result, it remains to be seen whether the current findings generalise to samples with a greater proportion of males and individuals of other gender identities.

### Implications and Conclusions

Taken together, existential concerns and FRP appear to be highly relevant to the migraine experience, highlighting some important clinical implications. Research on treatments for elevated illness-related fears has demonstrated that interventions such as cognitive-behavioural therapy (CBT) and supportive-expressive group therapy are effective in reducing FCR and FoP in people with cancer (Herschbach et al., 2010; Tauber et al., 2019). However, at present, no interventions have been evaluated in relation to FRP in migraine. The current results therefore underscore the need for the development and assessment of FRP interventions that can be applied to a greater range of illness groups.

The finding that all five existential concerns appeared equally associated with FRP in people with migraine suggest that each concern may each be an important treatment target. This implication is further supported by the finding that existential concerns partially mediated the relationship between disability and FRP. Existing psychological interventions for individuals with migraine (e.g. CBT, acceptance and commitment therapy; Dudeney et al., 2022) may benefit from specifically targeting all five existential concerns. Fortunately, these concerns have been shown to be amenable to treatment. One systematic review evaluating nine broad existential psychotherapies found some positive effects on existential well-being amongst people with advanced cancer (Terao and Satoh, 2021). More specifically, meaning-centred interventions have repeatedly been found to improve psychological outcomes in cancer patients (Breitbart et al., 2015; Kang et al., 2019). In addition, meta-analytic evidence has shown CBT is efficacious in reducing death anxiety (Menzies et al., 2018), albeit studies in samples with chronic illness are lacking. Although several treatment approaches show promise in alleviating existential concerns, further research and evaluation is needed to determine which therapeutic approaches are most effective for individuals with migraine who experience FRP.

## Data Availability

Data is available from the authors upon reasonable request.
